# Hybrid Films Prepared from a Combination of Electrospinning and Casting for Offering a Dual-Phase Drug Release

**DOI:** 10.3390/polym14112132

**Published:** 2022-05-24

**Authors:** Haoran Liu, Wenlai Jiang, Zili Yang, Xiren Chen, Deng-Guang Yu, Jun Shao

**Affiliations:** 1 School of Materials and Chemistry, University of Shanghai for Science and Technology, Shanghai 200093, China; 203613021@st.usst.edu.cn (H.L.); 203613110@st.usst.edu.cn (W.J.); 203613094@st.usst.edu.cn (Z.Y.); 2 Shanghai Institute of Technical Physics, Chinese Academy of Sciences, 500 Yutian Road, Shanghai 200083, China; xrchen@mail.sitp.ac.cn; 3 Shanghai Engineering Technology Research Center for High-Performance Medical Device Materials, Shanghai 200093, China

**Keywords:** electrospinning, casting, hybrid films, dual-phase release, nanofibers

## Abstract

One of the most important trends in developments in electrospinning is to combine itself with traditional materials production and transformation methods to take advantage of the unique properties of nanofibers. In this research, the single-fluid blending electrospinning process was combined with the casting film method to fabricate a medicated double-layer hybrid to provide a dual-phase drug controlled release profile, with ibuprofen (IBU) as a common model of a poorly water-soluble drug and ethyl cellulose (EC) and polyvinylpyrrolidone (PVP) K60 as the polymeric excipients. Electrospun medicated IBU-PVP nanofibers (F7), casting IBU-EC films (F8) and the double-layer hybrid films (DHFs, F9) with one layer of electrospun nanofibers containing IBU and PVP and the other layer of casting films containing IBU, EC and PVP, were prepared successfully. The SEM assessments demonstrated that F7 were in linear morphologies without beads or spindles, F8 were solid films, and F9 were composed of one porous fibrous layer and one solid layer. XRD and FTIR results verified that both EC and PVP were compatible with IBU. In vitro dissolution tests indicated that F7 were able to provide a pulsatile IBU release, F8 offered a typical drug sustained release, whereas F9 were able to exhibit a dual-phase controlled release with 40.3 ± 5.1% in the first phase for a pulsatile manner and the residues were released in an extended manner in the second phase. The DHFs from a combination of electrospinning and the casting method pave a new way for developing novel functional materials.

## 1. Introduction

Successful drug delivery needs supports from both pharmaceutical excipients, pharmaceutical techniques, and their effective combinations [1,2,3,4,5,6,7]. To a certain extent, the fields of pharmaceutics and drug delivery rely heavily on the inputs of materials sciences (new excipients) and engineering (new techniques) [8,9,10,11,12,13]. Thus, during the past half a century, on the one hand, more and more types of materials (including natural polymers, synthesis polymers, phospholipid, surfactants, and even inorganic materials) have been tried as new drug carriers for adjusting the drug release performances [14,15,16,17,18]. Among them, polymeric excipients are the main stream [19,20,21,22]. On the other hand, new materials processing and treatment methods, particularly nanotechnologies, are continuously explored for encapsulating drugs into pharmaceutical excipients for increasing their therapeutic effects [23,24,25,26].

**Figure 1:**
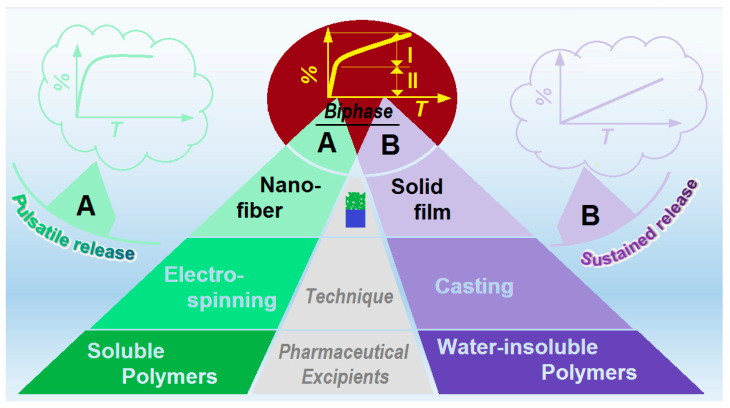
The strategies for preparing polymeric drug delivery systems for providing a dual-phase drug-controlled release.

Shown in Figure 1, on the left column, soluble polymers are frequently utilized to increase the fast dissolution and release of poorly water-soluble drugs, such as PVA, PVP, PEO, pullulan, gelatin, and so on [27,28,29]. When these polymers are transferred into nanofibers with a certain poorly water-soluble drug, the dissolution enhancement effects are greatly improved because of the unique properties of electrospun nanofibers, such as small diameters, high porosity, and the amorphous state of the loaded drug molecules [30,31]. Upon encountering water, the medicated nanofibers often are able to release the loaded drugs in a pulsatile manner, freeing the drug molecules all at once [32,33]. This drug controlled release profile is very useful for a series of diseases where fast therapeutic actions are required, e.g., fever, pain, depression, and heart disease [34,35,36].

In contrast, shown in the right column of Figure 1, water-insoluble and bio-degradable polymers are often exploited to prepare casting films for providing drug sustained release profiles [37,38,39,40,41]. As the polymeric carriers are insoluble in water, the loaded drug molecules must be freed into the dissolution medium through a diffusion mechanism [42,43,44,45]. This is also the dominant mechanism for many drug molecules released from their inorganic carriers, such as carbon nano tubes, silicon dioxide, and little molecules such as lipids [46,47,48,49]. The casting films often have a solid inner structure, which determines that the encapsulated drug molecules have a long way to diffuse for dissolution, and thus, a fine sustained release profile is achieved accordingly [50,51]. The drug sustained release profiles not only benefit the patients with reduced administration times being ensured, but also can keep a constant blood drug concentration, and thus, a safer therapeutic effect [52,53,54].

Both pulsatile and sustained releases profiles have their suitable scopes. However, the more sophisticated drug controlled release profiles of their combinations, i.e., biphasic or dual-phase release profiles, are able to manipulate the drug release in a more reasonable manner according to the biological rhythm [55]. For example, a pulsatile release at the initial time period for a quick reduction of inflammation and fever, and later, a second sustained release for keeping a constant blood drug concentration to maintain an efficacious therapeutic effect. However, the successful realization of this goal needs not only the applications of several pharmaceutical excipients, but also the advanced techniques or a combination of different kinds of techniques. For example, it is reported that core–shell nanofibers from coaxial electrospinning and Janus nanofibers from side-by-side electrospinning can provide these advanced drug controlled release profiles, with a fast release from the shell section or one side, and an extended release from the core section or another side [55,56]. However, these multiple-fluid electrospinning processes are more difficult to be scaled up and often need more skilled operation skills [57,58,59,60,61]. New facile method is highly desired for producing drug delivery systems that provide drug double-stage release.

Fortunately, one of the most important trends in the development of electrospinning is to combine itself with traditional materials production and transformation methods to take advantages of the unique properties of nanofibers [62,63,64,65,66,67,68]. Thus, in this research, a facile single-fluid blending electrospinning process was combined with the casting film method to fabricate a medicated double-layer hybrid for providing a dual-phase drug controlled release profile. Ibuprofen (IBU), a highly effective antipyretic and analgesic drug, and also a typical poorly water-soluble drug [69,70], was selected as a drug model. Two very common polymeric excipients, water-soluble polyvinylpyrrolidone (PVP) and water-insoluble ethyl cellulose (EC), were selected as drug carriers to show the combination of electrospinning and casting, and to offer a desired two-stage IBU release profile.

## 2. Experimental Section

### 2.1. Materials

Ibuprofen (IBU) was ordered from the Shanghai Macklin Biochemical Technology Co., Ltd. (Shanghai, China). Polyvinylpyrrolidone K60 (PVP K60, $M_{w}=$ 360,000), anhydrous ethanol, and dichloromethane (DCM) were obtained from Sigma-Aldrich Co. Ltd. (Shanghai, China). Ethyl cellulose (EC) was purchased from Sinopham Chemical Reagent Co. Ltd. (Shanghai, China). The water was doubly distilled before use, and all chemicals and regents used were analytical grade and were used directly.

### 2.2. Preparations and Optimizations

A series of solutions were prepared with various PVP concentrations from 2% to 4%, 5%, 6%, and 9% (*w/v* in anhydrous ethanol), which are denoted as F1, F2, F3, F4, and F5, respectively (Table 1). These samples were tested for determining the electrospinnable concentration limitation.

A homemade electrospinning apparatus was explored to implement the electrospinning processes. A high-voltage power supply (ZGF 60 kV/2 mA, Shanghai Sute Corp., Shanghai, China) and a syringe pump (KDS100, Core-Parmer, Vernon Hills, IL, USA) were exploited to provide the high voltage and to quantitatively drive the working fluids from a 20 mL syringe. The spinneret was a G21 stainless steel capillary (with inner and outer diameters of 0.51 and 0.82 mm, respectively). During all the experiments, the fluid flow rates and the collected distance of nanofibers were fixed at 1.5 mL/h, and 20 cm, respectively. The applied voltages were tuned between a range from 12.0 kV to 15.0 kV, on one hand, ensure the electrohydrodynamic processes, and on other hand, to prevent the droplets dripping from the nozzle directly. The ambient conditions were as follows: 22 ± 4 °C of temperature and 56 ± 5% of relative humidity.

As for the medicated nanofibers F6 and F7, one-third contents of IBU were added to the electrospinnable PVP solutions of 9% and 6% to form the blending fluids for preparing the IBU-loaded nanofibers F6 and F7, respectively. They are exploited to determine the influences of the added drug on the formations of nanofibers.

The casting film F8 of EC-IBU was prepared as follows: (1) 12.0 g EC and 2.0 g IBU were dissolved into a mixture of ethanol and DCM with a volume ratio of 50:50 by magnetic stirring for a clear solution; (2) the solution was treated using an ultrasonic instrument for 10 min, and later, 2.0 mL of it was poured into each glass Petri dish with a diameter of 12 cm; (3) these solutions were dried in a drying oven at 60 °C with air circulation until their weight was unchanged. Similarly, the casting film sections of the double-layer hybrid films F9 (DHFs) were prepared with one change, additional 2.0 g PVP being added in the blended solutions containing EC and IBU. Afterward, casting films were placed in a desiccator. For the preparation of electrospun mats comprising the sections of hybrid films F9, the applied voltage was fixed at 12 kV, and the feeding rates were fixed at 1.5 mL/h for 2 h. To ensure the integration of the two sections, a drop of ethanol was spread on the surface of the casting film, then the electrospun nanofibers were deposited on them.

### 2.3. Morphology

The morphologies of the samples from F1 to F9 was assessed using a scanning electron microscope (SEM, S-4800, Hitachi, Tokyo, Japan). The specimens were sputter-coated with gold under a nitrogen atmosphere to make them electronically conductive. Images were recorded at an excitation voltage of 20 ekV. The diameter distribution of fibers was estimated using the SEM machine directly. Before gold coating, the cross sections of DHFs F9 were prepared by placing several strips of them into liquid nitrogen for 20 min, and later, they were manually broken.

### 2.4. Physical Forms and Compatibility

An X-ray diffraction diffractometer (XRD) (D/Max-BR, RigaKu, Tokyo, Japan) was utilized to determine the physical forms of the medicated samples F7, F8, and F9. The test condition was over the 2θ range from 5° to 60° with a Cu Kα radiation at 40 mV and 30 mA. The raw material powders IBU, PVP, EC, samples F7, F8, and F9 were analyzed using Fourier transform infrared spectroscopy (FTIR) to examine the compatibility between the drug and the polymeric carrier. For FTIR tests, samples were prepared using the KBr pellet pressing method. An amount of 2.0 mg of each sample was dispersed within 200.0 mg of KBr medium by grinding and then compressed into transparent pellets under the pressure of 20.0 MPa. All pellets were then scanned with FTIR over the range of 4000 to 500 cm^−1^. Each spectrum was obtained using 8 scans with an accuracy of 2 cm^−1^.

### 2.5. In Vitro Dissolution Tests

The pure drug model IBU was dissolved in distilled water and prepared into a solution with a certain gradient concentration. The absorbance corresponding to the maximum absorption peak of the solution with different concentrations was measured using an ultraviolet spectrophotometer, and the standard equation of IBU was drawn according to the Lamber–Beer equation (Equation (1)):

(1) $$A=\mathrm{lg}[(I_{0}/I)]=\varepsilon LC$$

where *A* represents the absorbance,$\;I_{0}$ represents the intensity of the incident light, *I* represents the intensity of the emitted light, *ε* is the molar absorption coefficient of a substance, *L* is the optical path, and *C* is the concentration of the absorbent substance to be measured. According to the formula, it can be deduced that there is a one-to-one correspondence between the absorbance and the concentration of absorbed substances within the linear range. Therefore, a series of solutions of a certain concentration for testing the absorbance can be prepared. Then, a standard curve of the substance was established, and the equation could be used to further calculate the concentration of IBU in the tested samples.

The encapsulation efficiency (*EE*, %) of F7, F8, and F9 can be calculated using Equation (2), assessed as follows: an amount of 20.0 mg samples was placed into 50.0 mL ethanol to free all the loaded IBU. Then, 1.0 mL of the solution was dripped into 500 mL of distilled water to extract the IBU. After detection, the measured content of IBU (*C*_M_) can be calculated from the standard equation. The theoretical content of IBU (*C*_T_) of F9 can be calculated according to the experimental conditions using the following equation (Equation (3)):

*EE* (%)= *C*_M_/*C*_T_ × 100% (2)

(3) $$C_{T}\%=\frac{W_{e}\times C_{e-IBU}+W_{c}\times C_{c-IBU}}{W_{e}+W_{c}}$$

where *W* and *C* denote weight and content, respectively; the subscripts “*e*” and “*c*” refer to electrospun nanofibers and casting film, respectively; the compound subscripts “*e-IBU*” and “*c-IBU*” represent *IBU* contents in the solid products, which can be calculated from the preparation conditions.

The in vitro release characteristic curves of IBU from samples F7, F8, and F9 were determined according to the second paddle method in Chinese Pharmacopoeia Method II (2020 edition). In vitro dissolution conditions: 0.2 g of samples were put into the vessels holding 900 mL of stilled water. The dissolution parameters were set as 50 rpm and 37 ± 1 °C. At a predetermined timepoint, 5.0 mL samples were drawn and 5 mL of distilled water was injected to keep a constant dissolution volume. The absorbance of each sample was measured at λ_max_ = 260 nm using an ultraviolet spectrophotometer (Unico Co., Ltd., Shanghai, China). The accumulative release of IBU was calculated according to the following equation (Equation (4)). The results of these experiments are expressed as a mean value of percentage (*n* = 6):

(4) $$P(\%)=\frac{\rho_{n}\times V_{0}+\Sigma_{i=1}^{n-1}\rho_{i}\times V}{Q_{0}}\times 100\%$$

where *V*_0_ is the volume of the dissolved medium (mL), always 900 mL of distilled water; *V* is the volume of the sample taken from the dissolution media, always equal to 5 mL; *Q*_0_ is the total amount of IBU (mg) in 0.2 g samples; *ρ*_n_ is the measured concentration of the drug at the *n*-th time point (mg/L); *ρ*_i_ is the measured concentration of drug (mg/L) determined along the time points from the first one to the (*n* − 1)-th. 

## 3. Results and Discussion

### 3.1. Optimization of the Electrospinning Conditions

Electrospinning is facile for creating nanofibers due to a single-step straightforward process [71,72,73,74]. However, the optimization of electrospinning conditions for a continuous and robust preparation is not always an easy thing. This is because of the interrelationship of many parameters, such as exerted voltage, working distance, fluid rate, fluid’s properties, and also ambient conditions [75,76,77]. Among them, the concentration of the filament-forming polymer is the most important one, which will directly determine whether the processes are successful or not and what kinds of formats the final products have.

**Figure 2:**
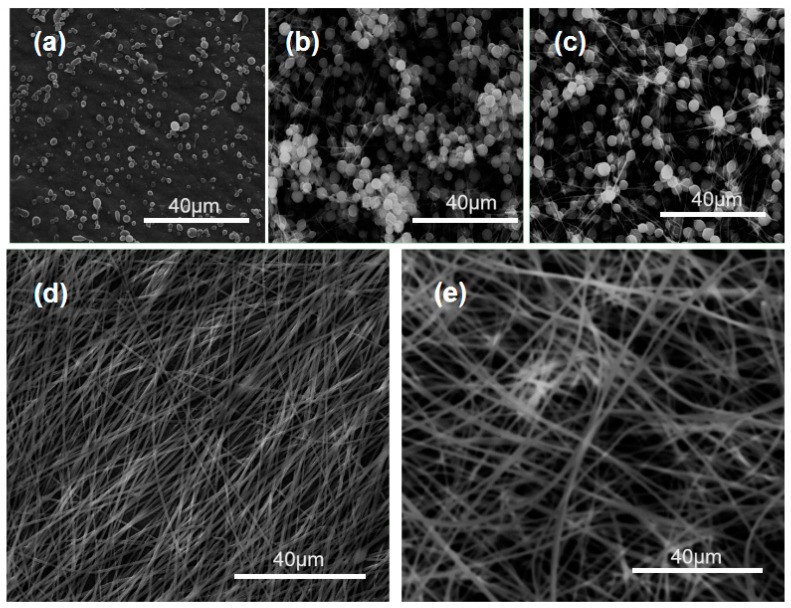
The studies about the electrospinnable concentrations of PVP: (**a**) F1—2%; (**b**) F2—4%; (**c**) F3—5%; (**d**) F4—6%; (**e**) F5—9%.

Shown in Figure 2 are SEM images of PVP K60 products prepared from its various concentrations. The trends are clear. The resultant products were electrosprayed particles of F1 and F2 when the PVP solutions had a concentration of 2% and 4%, respectively (Figure 2a,b). As the PVP concentration was increased to 5%, an obvious beads-on-a-string morphology occurred (Figure 2c). Further increases of PVP concentration to 6% and 9%, linear nanofibers (F5 and F6) without discerned beads or spindles were able to be achieved successfully (Figure 2d,e). By estimation, they had an average diameter of 0.54 ± 0.02 μm and 0.87 ± 0.04 μm, respectively.

**Figure 3:**
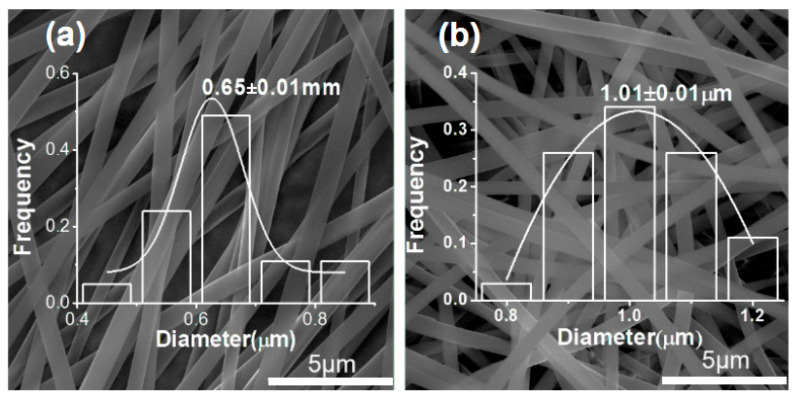
SEM images and diameter distribution of nanofibers with drug loading: (**a**) fibers F7 from a solution of 6% PVP and 3% IBU; (**b**) nanofibers F6 from a solution containing 9% PVP and 3% IBU.

When IBU (one-third weight of the PVP) was added into the polymeric solution, a blended working fluid containing both drug and carrier was formed. When the blended solutions experienced the electrospinning, the working processes were continuous and robust. The addition of IBU did not weaken the electrospinnability of PVP. Meanwhile, the corresponding nanofibers still showed a high quality, as indicated in Figure 3. Compared with the pure PVP nanofibers, the drug loaded nanofibers exhibited a slight increase in their diameters. F6 and F7 had a diameter of 1.01 ± 0.01 μm (larger than F5) and 0.65 ± 0.01μm (larger than F4). Thus, it can be concluded that the increase of polymer concentration would increase the diameter of the resultant nanofibers, and the addition of the drug can slightly increase the nanofibers’ size.

**Figure 4:**
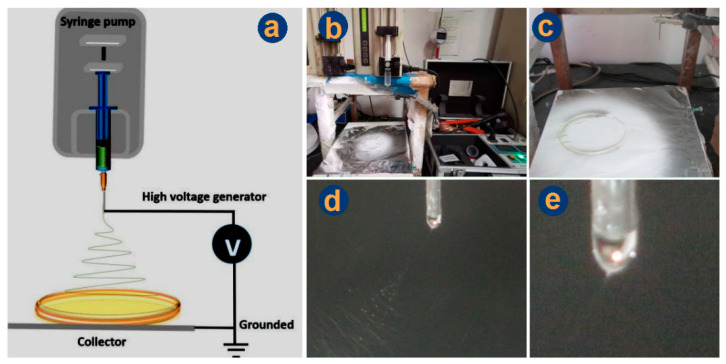
The combination of electrospinning and casting for preparing double-layer hybrid films: (**a**) a diagram showing the combination; (**b**) a digital photo about the whole system; (**c**) the collection of nanofibers by the glass, a Petri dish; (**d**) a typical electrospinning process; (**e**) the Taylor cone.

Based on the abovementioned results, nanofibers F7 were exploited to create the BHFs. The combination processes of electrospinning and casting are included in Figure 4. Figure 4a is the schematic diagram of the combination of the electrospinning system with casting, which is composed of four parts: high voltage generator, single axis spinneret (G21 metal capillary), injection pump, and collector. The only difference with the traditional electrospinning system lies in the fact that a casting film was directly utilized to collect nanofibers. Figure 4b shows the overall appearance of the working apparatus and Figure 4c is a magnified image of the collection. It can be clearly seen that some of the electrospun nanofibers were deposited on the casting film. Some nanofibers were scattered on the aluminum foils. This situation can be improved when production on a large scale is developed for creating the DHFs. Under the optimized condition, a typical working process was recorded in Figure 4d, in which the straight fluid jet and the gradual increase of bending and whipping circles are obvious. The initial Taylor cone hung on the nozzle of the spinneret is given in Figure 4e. These phenomena had no difference with the traditional ones.

### 3.2. Morphology and Janus Structure

**Figure 5:**
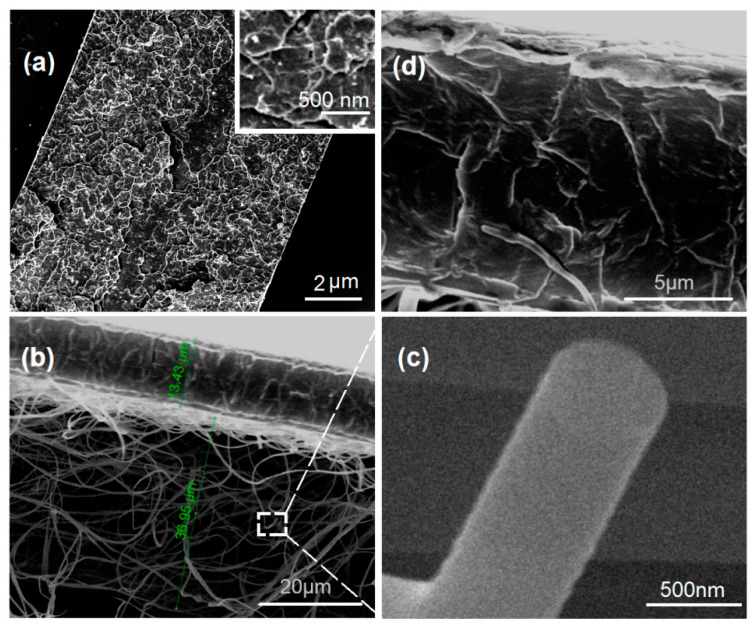
The SEM cross-sections of products: (**a**) casting film F8; (**b**) the DHFs of F9; (**c**) an enlarged image of a nanofiber’s cross-section; (**d**) an enlarged image of the casting film section of DHFs F9.

The SEM images of the casting films F8 and the DHFs F9 are included in Figure 5. The cross-sections of F8 are solid and compact (Figure 5a). However, there are some particles on it, as indicated by white dots in the above-right inset. This gives a hint that some of the IBU molecules might re-crystallize to result in a solid phase separation from the cast IBU-PVP films. Figure 5b is a full image of the DHFs F9, in which the casting layer and the electrospun mat have a thickness of 13.43 and 36.95 μm, respectively. An enlarged image of the cross-section of PVP-IBU nanofibers is given in Figure 5c, from which it is clear that no discerned phase separation occurred on both the surface and also the cross-section. Similarly, no discerned particles can be found on the cross-section of the casting layer of F9, as indicated by Figure 5d. Compared with F8 containing IBU and EC, the addition of PVP in the casting layer of F9 generated the differences.

### 3.3. Physical State and Compatibility

**Figure 6:**
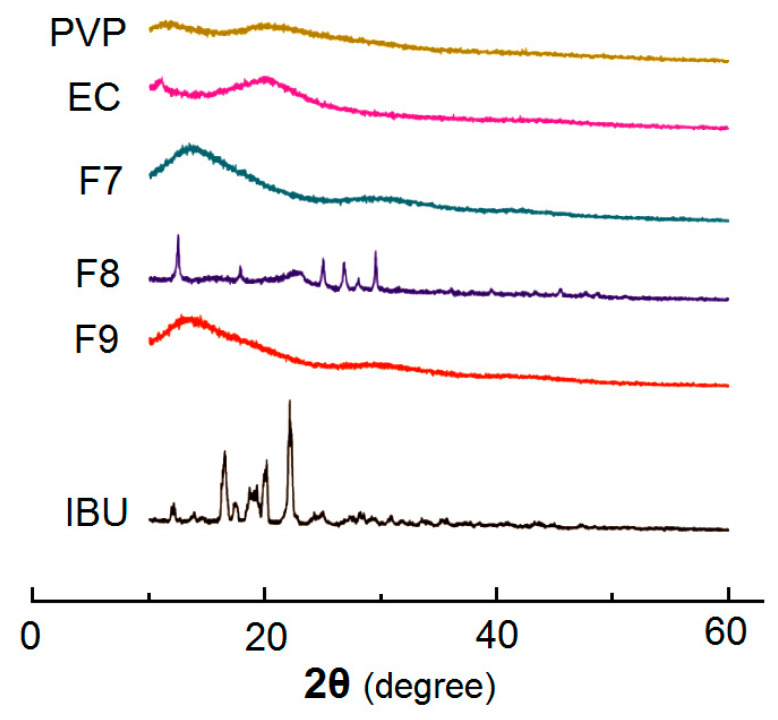
XRD patterns of PVP, EC, IBU and their products F7, F8, and F9.

The XRD patterns of the samples are included in Figure 6. The pure drug IBU powders (white color with a diameter smaller than 75 μm) show many sharp peaks. The characteristic diffraction peaks appear at diffraction angles 2θ of 6.10°, 12.21°, 16.58°, and 22.28°, indicating the raw IBU being presented in a crystal form. In contrast, the patterns of EC and PVP have no any sharp peaks but wide humps, which indicate that the two polymers exist in an amorphous form. Nanofibers F7 is the medicated sample made by electrospinning, and it can be seen that the sharp peak of IBU disappeared, indicating that PVP in the nanofibers F7 interacted with the IBU to form an amorphous PVP-IBU nanocomposites. Sample F8 was prepared by the casting methods. There are obvious small peaks above the baselines in the patterns, indicating the presence of crystalline materials. The formation of casting film needs a relatively long time period. During the drying processes, the IBU molecules might re-crystallize into particles to generate solid phase separation, which is also suggested by the SEM images in Figure 5a. The patterns of the DHFs F9 show only one broad halo without any discerned peaks, giving a hint that no IBU re-crystallization phenomenon occurred during the preparation processes of casting layers in them. The addition of PVP could significantly improve the compatibility of IBU with the carriers.

**Figure 7:**
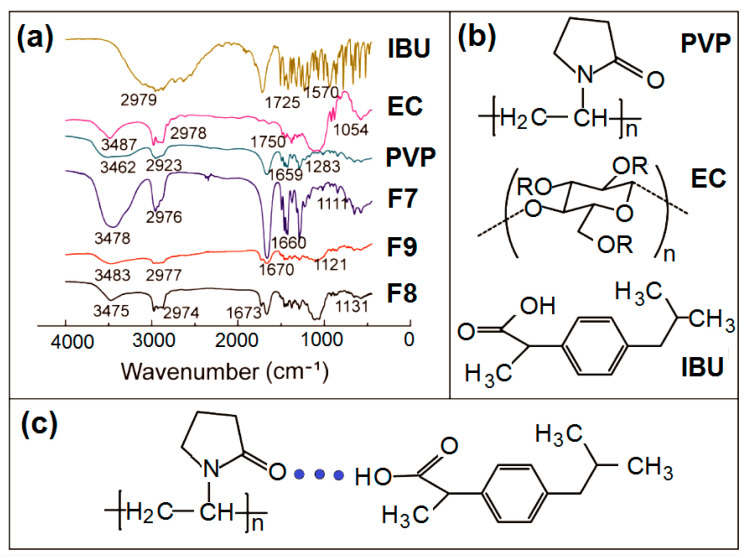
Compatibility studies: (**a**) FTIR spectra of PVP, EC, IBU and their products F7, F8, and F9. (**b**) The molecular formula of the raw materials; and (**c**) the hydrogen bonding between IBU and PVP molecules.

The FTIR spectra of the PVP, EC, IBU and their products F7, F8, and F9 are included in Figure 7. There are -C=O groups in both IBU and PVP molecules (Figure 7b), which vibrate at 1725 cm^−1^ and 1659 cm^−1^ (Figure 7a), respectively. Meanwhile, there are -OH groups in the IBU molecules. Thus, hydrogen bonding can be easily formed between IBU and PVP to increase their compatibility, which is shown in Figure 7c. The spectra of nanofibers F7 and DHFs F9 obviously verified these deductions. Most of the sharp peaks in the spectra of IBU have decreased or even totally disappeared from the spectra of F7 and F9. As for the spectra of F8, the signals from IBU also decreased to a large extent. At least IBU can interact with IBU through hydrophobic interactions between the benzene rings of IBU and long carbon chains of EC although they are weaker than the hydrogen bonding of IBU-PVP.

### 3.4. In Vitro Drug Release Profiles

The 2 mL casting solution formed a solid film with a weight of 0.327 ± 0.041 g (*n* = 10) on the 12 cm Petri dish. The 2 h of 1.5 mL/h electrospinning added a weight of 0.134 ± 0.008 g (*n* = 10) medicated nanofibers on the casting films. Thus, the density of the casting layer (ρ_c_) and electrospun nanofiber layer (ρ_e_) could be also calculated according to equation 5 and 6 (Equations (5) and (6)).

Ρ_c_ = W_c_/V_c_ = 0.327/(6 × 6 × π × 0.00134) = 2.15 g/cm^3^ (5)

ρ_e_ = W_e_/V_e_ = 0.114/(6 × 6 × π × 0.00370) = 0.27 g/cm^3^ (6)

Both casting and electrospinning are methods based on the evaporation of solvents. Thus, the EE% should be 100% provided the encapsulated drugs have no physical properties of volatilization or sublimation and are chemically stable. Here, the determined EE% for F7, F8, and F9 are 97.6%, 101.5%, and 101.3%, respectively (Table 2). The 2 h electrospinning should produce nanofibers (2 × 1.5) × (2% + 6%) = 0.270 g. The collected nanofibers on the DHFs was 0.114 g, thus, only 42.2% of the electrospun nanofibers were successfully converted into DHFs. However, this issue can be easily solved in large-scale production because no Petri dishes are needed.

**Figure 8:**
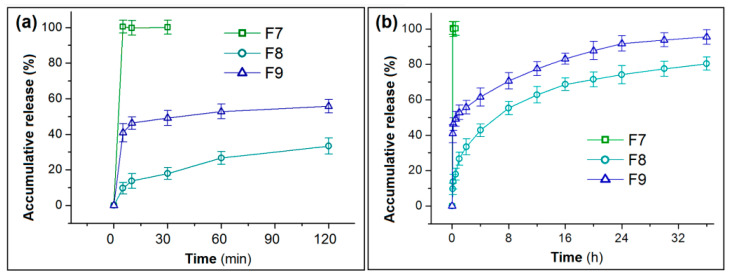
In vitro release profiles of IBU: (**a**) data of the first 2 h; (**b**) data of the full time period of 36 h.

According to the weight of collected nanofibers and the casting layer in the DHFs F9, the theoretical release content ratio in the two stage should be {0.114 × [2%/(2% + 6%]}:{0.327 × [2%/(2% + 2% + 12%]} = 0.0285:0.0409, i.e., the pulsatile release in the first stage should be 41.1%, theoretically. In the measurement, the accumulative release of F9 in the initial 5 min was 40.3 ± 5.1%, being close to the theoretical value. In comparison, the nanofibers F7 released 100.5 ± 3.7% at the same time period, i.e., releasing all the drug in a pulsatile manner. The casting film F8 released 9.6 ± 3.2% of the loaded IBU (Figure 8a). Thus, the DHFs effectively increased the release amount of IBU at the beginning, which is favorable for eliminating symptoms of disease rapidly.

After 36 h in vitro dissolution tests, F8 and F9 released 80.3 ± 4.1% and 95.4 ± 4.7% of the loaded IBU (Figure 8b). The solid EC-IBU film F8 could not release all the loaded IBU because that EC is totally insoluble in water. Thus, a small portion of IBU was totally enclosed by EC molecules. The water molecules could not reach them to free them to the dissolution media. In contrast, the DHFs F9 were able to release over 95% of the loaded IBU. The addition of PVP in the casting layer should play its important role in promoting the exhaustion of IBU.

**Figure 9:**
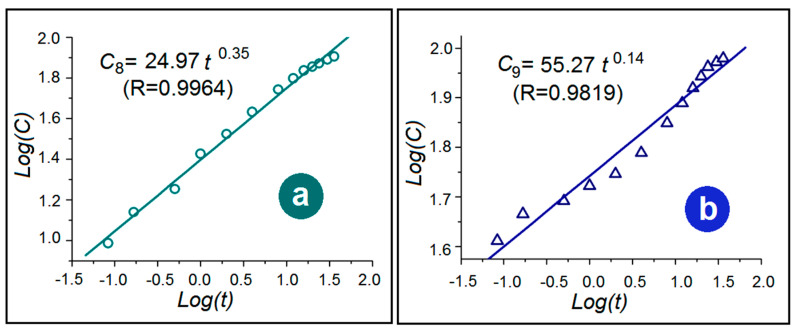
The drug release kinetics from the films of F8 (**a**) and F9 (**b**).

Peppas’ equation (Equation (7)) is exploited to disclose the kinetic behaviors of drug molecules when they were released from casting films F8 (Figure 9a) and the DHFs (Figure 9b).

*C* = *k*
× t ^n^ (7)

where *C* represent the accumulative release in percentage, *k* is a constant, and the value of exponent n can be exploited to indicate the drug release kinetic mechanism [78]. For F8 and F9, the equations are *C*_8_ = 24.97 t ^0.35^ (R = 0.9964) and *C*_9_ = 55.27 t ^0.14^ (R = 0.9819), respectively. The n values of 0.35 (˂0.45) and 0.14 (˂0.45) indicate that both F8 and F9 released the loaded IBU in the casting films in a typical diffusion manner.

### 3.5. The Dual-Phase Release Mechanism from the DHFs

**Figure 10:**
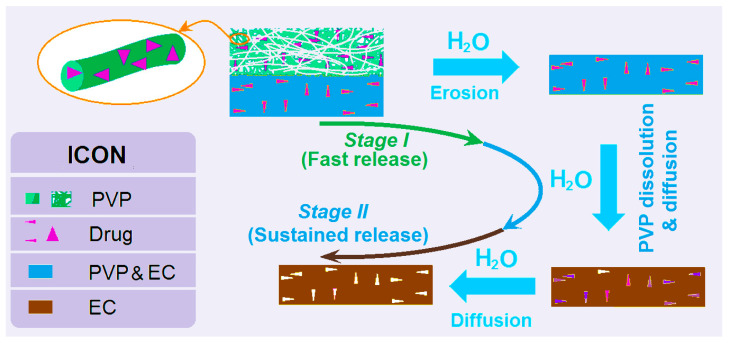
Mechanism diagram of dual-phase release from the DHFs F9.

A diagram shows the mechanism by which the DHFs F9 are able to provide the dual-phase drug controlled release profiles (Figure 10). The PVP nanofiber layer can release the loaded drug in a pulsatile way because of the following reasons: (1) PVP is a soluble polymeric excipient; (2) the drug IBU distributes all over the PVP matrix in an amorphous state; (3) the unique properties of the nanofibers, which include small diameter, large surface area, and huge porosity. When encountering water, the PVP nanofibers will be dissolved all at once, and correspondingly, the loaded IBU will be free into the water through the erosion of PVP.

Later, the casting layer will gradually release the loaded IBU but in a different manner. Firstly, the PVP molecules distributed within the EC matrix will be released to form passageways for the water molecules to diffuse into the insoluble solid EC films and thus facilitate the IBU molecules to be free out to the dissolution media. Both PVP and IBU molecules are released through the diffusion mechanism, but the PVP molecules will promote the diffusion of both water and IBU molecules, and finally, the release of IBU molecules through a typical diffusion mechanism.

As a hot nano technique in scientific research [79], electrospinning is presently approaching commercial applications in a wide variety of fields. Among them, biomedical applications hold the most promise. Particularly, the medical applications of different kinds of medical accessories and possible tissue engineering products are underway urgently [80,81]. The present hybrid films from a combination of electrospinning and casting should be candidates for them. Certainly, the facile production of a large scale of electrospun nanofibers will pave the possible ways for realizing this goal [82,83].

## 4. Conclusions and Perspective

With IBU as a common model of a poorly water-soluble drug, and EC and PVP K60 as the polymeric excipients, electrospun medicated IBU-PVP nanofibers (F7), casting IBU-EC films (F8), and DHFs (F9) with one layer of electrospun nanofibers containing IBU and PVP and the other layer of casting films containing IBU, EC, and PVP, were prepared successfully. The SEM images clearly demonstrated that F7 were in linear morphologies without beads or spindles, F8 were solid films, and F9 consisted of one porous fibrous layer and one solid layer. XRD and FTIR results verified that both EC and PVP are compatible with IBU. In vitro dissolution tests indicated that F7 were able to provide a pulsatile IBU release, F8 offered a typical drug sustained release, whereas F9 were able to exhibit a dual-phase controlled release. The mechanism that the DHFs provided the dual-phase drug controlled release is disclosed. The DHFs from a combination of electrospinning and casting method pave a new way for developing novel functional materials.

Based on the reasonable selections of polymers with different properties, the present study shows a strategy for the combination of electrospinning with a traditional casting method for producing functional hybrid films. In this way, there should be many similar possibilities for further investigations. For example, from a standpoint of technique combination, 3D printing, as a flexible method in creating complex micro structures within the printed parts [84,85,86,87], can be combined with both electrospinning and casting for generating novel polymeric hybrids. From a standpoint of tailoring polymer carriers, insoluble inert polymers can be integrated with biodegradable polymers for manipulating drug release behaviors [88]. From a standpoint of drug delivery applications, the present hybrid films can be explored for many similar active ingredients that dual-phase release can ensure a better therapeutic effect. Certainly, the two-layer films can be loaded with different kinds of drugs for a combination therapy, which is presently very popular in treating cancers.

## Figures and Tables

**Table 1:** Data for preparations and optimizations.

Denoted	Technique	Working Fluid (*w/v*%)	Products
F1	Electro- spraying	2% PVP	Particles
F2		4% PVP	Particles
F3		5% PVP	Beads-on-a-string
F4	Electro- spinning	6% PVP	Nanofibers
F5		9% PVP	Nanofibers
F6		9% PVP & 3% IBU	Nanofibers
F7		6% PVP & 2% IBU	Nanofibers
F8	Casting	12% EC + 2% IBU	Solid films
F9	Electrospinning//Casting	2% IBU + 6% PVP//2% IBU + 2% PVP + 12% EC	Double-layer hybrid films (DHFs)

**Table 2:** Drug content in different prepared nanofibers (*n* = 3).

No.	Process	C_T_ (wt%) ^a^	C_D_ (wt%) ^b^	*EE* (%)
F7	Electrospinning	25	25.62 ± 0.25	97.6%
F8	Casting	12.5	12.31 ± 0.18	101.5%
F9	Combination	15.80	16.01 ± 0.13	101.3%

^a^ Theoretical drug content; ^b^ Detected drug content.

## Data Availability

The data supporting the findings of this manuscript are available from the corresponding authors upon reasonable request.
